# Sympathoinhibitory electroacupuncture (EA) interacts positively with anti-inflammatory EA alleviating blood pressure in hypertensive rats

**DOI:** 10.3389/fcvm.2023.1140255

**Published:** 2023-05-30

**Authors:** Liang-Wu Fu, Yiwei D. Gong, Anh T. Nguyen, Zhi-Ling Guo, Stephanie C. Tjen-A-Looi, Shaista Malik

**Affiliations:** Susan Samueli Integrative Health Institute, College of Health Sciences, University of California-Irvine, Irvine, CA, United States

**Keywords:** hypertension, Acupuncture, sympathetic activity, Inflammatory mediator, positive interaction

## Abstract

Elevated sympathetic activity and chronic inflammation are known contributory factors observed in hypertension. We have observed that sympathoinhibitory electroacupuncture (SI-EA) at acupoints ST36-37 alleviates sympathetic activity and hypertension. Additionally, EA at acupoints SP6-7 exerts anti-inflammatory (AI-EA) effects. However, it is not known whether simultaneous stimulation of this combination of acupoints attenuates or enhances individual effects. A 2 × 2 factorial design was used to test the hypothesis that combining SI-EA and AI-EA (cEA) leads to greater reduction of hypertension by decreasing sympathetic activity and inflammation in hypertensive rats than either set of acupoints alone. Dahl salt-sensitive hypertensive (DSSH) rats were treated with four EA regimens including cEA, SI-EA, AI-EA, and sham-EA twice weekly for five weeks. A group of normotensive (NTN) rats served as control. Systolic and diastolic BP (SBP and DBP) and heart rate (HR) were measured non-invasively by tail-cuff. Plasma norepinephrine (NE), high-sensitivity C-reactive protein (hs-CRP) and interleukin 6 (IL-6) concentrations were determined with ELISA at the completion of treatments. DSSH rats on high salt diet progressively developed moderate hypertension within five weeks. DSSH rats treated with sham-EA showed continuous increase in SBP and DBP and elevations in plasma NE, hs-CRP, and IL-6 levels relative to NTN control. Both SI-EA and cEA decreased SBP and DBP, and had corresponding changes in biomarkers (NE, hs-CRP, and IL-6) compared with sham-EA. AI-EA prevented SBP and DBP elevation and decreased IL-6 and hs-CRP relative to sham-EA. Importantly in DSSH rats that received repetitive cEA treatment, SI-EA interacted positively with AI-EA leading to greater reduction of SBP, DBP, NE, hs-CRP, and IL-6 than SI-EA or AI-EA alone. These data suggest that by targeting both elevated sympathetic activity and chronic inflammation, cEA regimen results in a greater reduction of BP effects in treating hypertension compared to using individual SI-EA or AI-EA alone.

## Introduction

Hypertension (HTN) affects approximately one billion individuals worldwide and involves multiple pathways, including the autonomic nervous and immune systems (1–3). Despite seven classes of antihypertensive drugs available, nearly 50% of hypertensive patients still do not have their blood pressure (BP) under control (4–6). Uncontrolled HTN is a major risk factor for severe cardiovascular diseases including stroke, myocardial infarction, and heart failure. The reasons for inadequate treatment and BP control are complicated. Current antihypertensive medications do not address the sympathoexcitatory and inflammatory mechanisms underlying the development and progression of hypertension (7–9). Novel and effective pharmacological and non-pharmacological therapies to optimize BP control would fill a major gap in current available treatment options. Acupuncture has been recommended by WHO as an alternative therapy for the management of HTN (10). Previous clinical trials and preclinical studies have examined effects of acupuncture in lowering BP in hypertensive subjects by utilization of numerous and variable acupoints such as CV4, CV6, CV8, CV10, GB20, GB34, GV20, LI4, LI11, LR2, LR3, PC6, SP6, ST8, ST36, and ST40 with varying outcomes (11–16). We have conducted a series of mechanistic and clinical studies focusing on specific acupoints (e.g. P5-6 and ST36-37) and demonstrated the role of stimulating these specific acupoints with electroacupuncture (EA) in reducing elevated sympathetic activity and alleviating elevated BP (13, 17, 18). EA is a form of acupuncture that uses electrical stimulation of the needles inserted into the acupoints. In traditional acupuncture, needles are inserted into acupoints and stimulated manually. Traditional acupuncture involves individualized therapy where the practitioner stimulates a combination of acupoints based traditional assessments.

We have shown for over two decades in numerous studies the neuronal pathways and circuitry, the specific neurotransmitter systems, and mRNA and c-Fos expressions involved in the actions of EA through the stimulation of somatosensory nerve fibers with defined parameters (17, 19–27). Stimulation of the somatosensory nerve fibers for 30 min decreases the activity of presympathetic cardiovascular neurons in the rostral ventral lateral medulla (rVLM), and paraventricular nucleus (PVN), and sympathetic outflow and consequently, reduction of the elevated BP (19, 28–30). We have shown pathways and circuitry spanning from the hypothalamus to the medulla such as the arcuate nucleus (ARC), ventrolateral periaqueductal gray (vlPAG), nucleus raphe pallidus (NRP), rVLM and PVN participating in the sympathoinhibitory actions of EA on elevated BP in hypertensive pre-clinical and clinical studies (19, 23–27, 31).

Hypertensive animal models including Dahl salt-sensitive hypertensive (DSSH) and spontaneously hypertensive rats (SHR) display not only elevated sympathetic nerve activity (SNA) but also chronic inflammation (22, 32–34). Increased chronic inflammation indicated by elevated plasma high sensitive C-reactive protein (hs-CRP) and/or pro-inflammatory cytokines have been shown in DSSH, SHR, and angiotensin-II induced hypertensive rats (35–38). Pro-inflammatory cytokine specifically interleukin-6 (IL-6) is elevated in subjects with hypertension (9). We have demonstrated that EA stimulation at acupoints ST36-37 overlying the deep peroneal nerve reduces reflex elevated and sustained high blood pressure in hypertensive subjects including patients and animal models by decreasing the elevated sympathetic outflow, now termed as sympathoinhibitory EA (SI-EA) (22, 26, 29, 30). Others have reported that EA at SP6-7 acupoints overlying the tibial nerve suppresses inflammation, named as anti-inflammatory EA (AI-EA) (39, 40). However, it is unclear if AI-EA contributes to the reduction of BP in hypertensive subjects. Also, more importantly, it is unknown if combining SI-EA and AI-EA (cEA) therapy targeting both downstream sympathetic and immune systems results in greater BP reductions in hypertensive subjects than either SI-EA or AI-EA alone.

The present study was performed to test the hypothesis that cEA therapy leads to a greater BP reduction in hypertensive rats by decreasing both elevated SNA and inflammation than SI-EA or AI-EA alone with a 2 × 2 factorial design. With this design, we can examine the interaction between these two sets of acupoints.

## Results

### Changes in BPs and HRs in DSS rats in the development of hypertension

**Figure 1:**
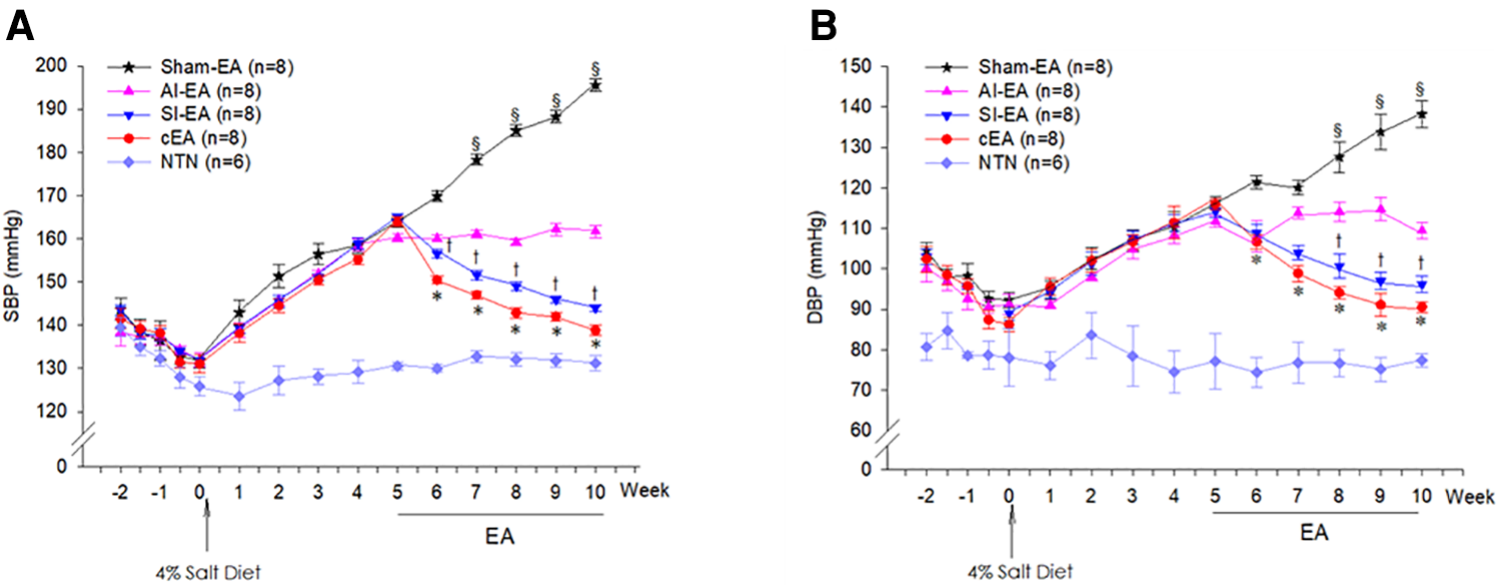
Dynamic or weekly changes of systolic blood pressure (SBP, panel **A**) and diastolic blood pressure (DBP, panel **B**) in the Dahl salt-sensitive hypertensive (DSSH) rats treated with repetitive electroacupuncture (EA) and in DSS normotensive (NTN) rats. The BPs at week 5 were defined as pre-EA levels in the 4 groups of rats treated with EA regimens. The 4 EA regimens include sympathoinhibitory EA (SI-EA), anti-inflammatory EA (AI-EA), combined SI-EA and AI-EA (cEA), and sham-EA. Data are expressed as mean ± SE. §, *P* < 0.05, post-sham EA vs. pre-sham EA; †, *P* < 0.05, post-SI-EA vs. pre-SI-EA; *, *P* < 0.05, post-cEA vs. pre-cEA. Note: BP data were recorded twice per week from −2 to 0 week (see Supplementary Material).

Following a five weeks 4% salt diet, the SBP and DBP significantly increased respectively from 131.8 ± 0.7 mmHg and 90.2 ± 1.0 mmHg at week 0 to 163.3 ± 0.6 mmHg and 114.3 ± 0.9 mmHg at week 5 (Figure 1), indicating that all 32 Dahl salt-sensitive (DSS) rats developed moderate hypertension. The SBP and DBP of 6 other DSS rats on standard chow remained in normotensive range from week 0 to week 5, serving as normotensive controls. As expected, there were no significant changes in HRs throughout the 5 weeks and among the hypertensive and normotensive DSS rats (Supplementary Figure S1).

### Effects of SI-EA, AI-EA, and cEA on BP in DSS hypertensive rats

The 32 hypertensive DSS rats were allocated into 4 EA regimen groups and subjected to twice-weekly repetitive EA treatments for 5 weeks starting from week 5. We defined the hypertensive SBP and DBP levels at week 5 as pre-EA BPs in four EA treated groups. At week 10, we observed that the SBP significantly reduced from 164.0 ± 1.1 to 138.9 ± 1.2 mmHg and DBP reduced from 116.1 ± 1.3 to 92.1 ± 1.3 mmHg in response to 5 weeks of cEA treatment (P < 0.05). The SBP significantly reduced from 165.1 ± 0.7 to 144.0 ± 0.8 mmHg and DBP reduced from 115.0 ± 2.3 to 97.1 ± 2.1 mmHg in response to 5 weeks of SI-EA (P < 0.05, Figure 1). However, both SBP and DBP remained in the pre-EA range (SBP, 160.2 ± 0.8 mmHg and DBP, 109.7 ± 1.8 mmHg) in response to 5 weeks of AI-EA treatment. Conversely, at week 10, SBP significantly increased from 164.2 ± 1.5 to 195.6 ± 1.5 mmHg and DBP increased from 116.2 ± 1.2 to 138.1 ± 3.3 mmHg in response to 5 weeks of sham-EA (Figure 1). Compared to sham-EA, all EA treatments including cEA, SI-EA and AI-EA prevented further increase of SBP and DBP (P < 0.05, Figure 1) from week 5 (at the pre-EA levels) to week 10.

**Figure 2:**
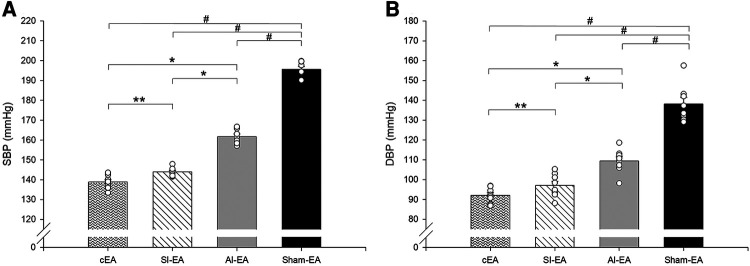
Bar histograms showing systolic blood pressure (SBP, panel A) and diastolic blood pressure (DBP, panel B) at end of week 10 in rats treated with cEA, SI-EA, AI-EA and sham-EA. #, *P* < 0.05, active EA vs. sham-EA; *, *P* < 0.05, cEA or SI-EA vs. AI-EA; and **, *P* < 0.05, cEA vs. SI-EA.

Furthermore, we observed that in DSSH rats treated with cEA, the combination of SI-EA with AI-EA resulted in greater reductions in SBP and DBP than SI-EA alone or AI-EA alone (Table 1). There was a positive interaction between SI-EA and AI-EA. The effect of AI-EA was only noticeable when it was part of the cEA treatment and not when used alone (P < 0.05 for interaction effect). Specifically, in pair-wise comparison, we observed greater reduction of SBP in DSSH rats that received cEA treatment compared to the SI-EA treatment (138.9 ± 1.2 vs. 144.0 ± 0.8 mmHg, P < 0.05, cEA vs. SI-EA, Figure 2) or compared to the AI-EA (138.9 ± 1.2 vs. 161.7 ± 1.3 mmHg, P < 0.05, cEA vs. AI-EA, Figure 2). We also observed greater reduction of DBP in rats with the cEA than the SI-EA (92.1 ± 1.3 vs. 97.1 ± 2.1 mmHg, cEA vs. SI-EA) or the AI-EA (92.1 ± 1.3 vs. 109.4 ± 2.1 mmHg, cEA vs. AI-EA, Figure 2) alone. As expected, Heart rates were not influenced by EA in all 4 groups of rats (Supplementary Figure S1).

**Table 1:** Blood pressure response (at week 10) following 5 weeks of repetitive SI-EA, AI-EA, cEA (i.e., SI-EA plus AI-EA), and sham-EA treatment in DSSH rats.

Δ SBP (mmHg)				
		SI-EA		Margin
		Yes	No	
AI-EA	Yes	−25.1 ± 1.6*^,^**	3.7 ± 1.4*	−10.9 ± 3.8
	No	−21.0 ± 1.3*	31.4 ± 1.9	5.2 ± 6.9
Margin		−23.1 ± 1.1	17.3 ± 3.8	
Δ DBP (mmHg)				
AI-EA	Yes	−24.0 ± 1.5*^,^**	−0.3 ± 1.2*	−12.1 ± 3.2
	No	−17.9 ± 1.4*	21.9 ± 3.5	2.0 ± 5.5
Margin		−21.0 ± 1.3	10.8 ± 3.4	

Values are means ± SE. **Δ**, change; SBP, systolic blood pressure; DBP, diastolic blood pressure. **Δ** SBP is equal to the change in SBP at week 10 minus SBP at week 5. **Δ** DBP is equal to DBP at week 10 minus DBP at week 5. Two-way ANOVA analysis indicates that SI-EA interacts positively with AI-EA in lowering SBP and DBP (*P* < 0.05).

* *P* < 0.05, compared to sham-EA.

**P < 0.05, cEA compared to AI-EA or SI-EA.

### Weekly changes of SBPs and DBPs in DSSH rats during 5-week EA treatment course

During the 5-week sham-EA treatment course, both SBP and DBP significantly increased from week 7 to week 10 compared to the pre-EA BPs at week 5 (Figure 1, Supplementary Figure S2). In contrast, during both SI-EA and cEA treatment courses, SBPs and DBPs significantly decreased during each successive week starting from week 6 compared to the pre-EA BPs level (Figure 1, Supplementary Figure S2). In addition, we observed that SBP and DBP did not change significantly during 5-week AI-EA treatment relative to their pre-EA BPs (Figure 1, Supplementary Figure S2), indicating that AI-EA fails to lower BPs below the levels observed before AI-EA treatment but prevented BPs from increasing. Heart rates in the four EA groups and normotensive control group did not alter significantly during the 13-weeks experimental course (Supplementary Figure S1).

### Plasma biomarker changes of DSSH rats in response to SI-EA, AI-EA, and cEA

#### Plasma NE levels

**Figure 3:**
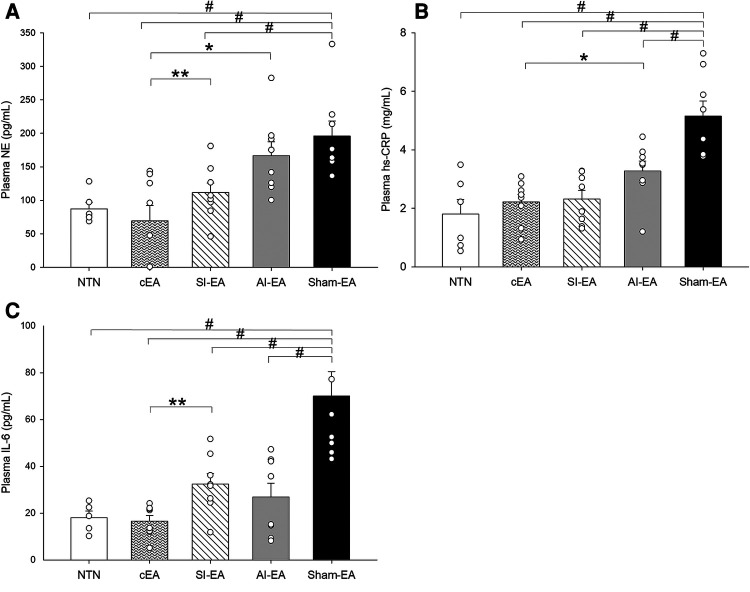
Plasma biomarker concentrations are increased in the Dahl salt-sensitive hypertensive rats treated with sham electroacupuncture (Sham-EA) compared to normotensive (NTN) rats. Plasma norepinephrine (NE) (**A**) is reduced in cEA and SI-EA treated rats compared to Sham-EA rats. Inflammatory biomarkers (hs-CRP (**B**) and IL-6 (**C**) are reduced in each EA treated (SI-EA, AI-EA, and cEA) groups of rats compared to Sham-EA rats. Data are expressed as mean ± SE. #, *P* < 0.05, each group compared to sham-EA; *, *P* < 0.05 AI-EA vs. cEA; **, *P* < 0.05, SI-EA vs. cEA.

At the end of the study, the plasma NE concentration was significantly higher in the sham-EA group compared to normotensive group (Figure 3A). After 5 weeks of repetitive SI-EA or cEA treatment, the plasma NE level in DSSH animals was significantly lower than in the sham-EA group. However, repetitive AI-EA treatment did not reduce the NE level compared to sham-EA group. We did not observe an additional reduction in the concentration of NE in the cEA-treated group compared to the SI-EA group (Table 2).

**Table 2:** Concentrations of NE following 5 weeks of repetitive SI-EA, AI-EA, cEA (i.e., SI-EA plus AI-EA), and sham-EA treatments in DSSH rats.

NE (pg/mL)				
		SI-EA		Margin
		Yes	No	
AI-EA	Yes	69.4 ± 22.7*^,^**	167 ± 20.7	118 ± 19.4
	No	112 ± 14.4*	196 ± 22.4	154 ± 16.9
Margin		90.4 ± 14.1***	181 ± 15.2	

Values are means ± SE. NE, Norepinephrine. Two-way ANOVA analysis indicates no interaction between SI-EA and AI-EA in lowering NE concentration.

* *P* < 0.05, compared to sham-EA.

** *P* < 0.05, cEA compared to AI-EA or SI-EA.

*** *P* < 0.05, All SI-EA treated rats (cEA and SI-EA groups) compared to Non-SI-EA animals (AI-EA and sham-EA groups).

#### Plasma hs-CRP levels

Plasma hs-CRP concentration in the sham-EA treated rats was higher than normotensive rats (*P* < 0.05) as shown in Figure 3B. The hs-CRP levels in the three groups of DSSH rats treated with SI-EA alone, AI-EA alone and cEA were significantly lower than the sham-EA group. Furthermore, SI-EA interacted positively with AI-EA resulting in a greater reduction in hs-CRP than AI-EA alone (*P* < 0.05 for interaction effects, Table 3).

**Table 3:** Concentrations of hs-CRP after 5 weeks of repetitive SI-EA, AI-EA, cEA (i.e., SI-EA plus AI-EA), and sham-EA treatment in DSSH rats.

hs-CRP (mg/ml)				
		SI-EA		Margin
		Yes	No	
AI-EA	Yes	2.22 ± 0.26*^,^**	3.27 ± 0.35*	2.74 ± 0.25
	No	2.32 ± 0.30*	5.16 ± 0.51	3.74 ± 0.46
Margin		2.27 ± 0.19	4.21 ± 0.38	

Values are means ± SE. Hs-CRP, high-sensitivity C-reaction protein. Two-way ANOVA analysis indicates that SI-EA interacts positively with AI-EA in lowering hs-CRP (*P* < 0.05).

*P < 0.05, compared to sham-EA.

**P < 0.05, cEA compared to AI-EA or SI-EA.

#### Plasma IL-6 levels

Plasma IL-6 concentration in the DSSH rats treated with sham-EA was higher than normotensive rats (*P* < 0.05, Figure 3C). The IL-6 levels in the three groups of DSSH rats treated with SI-EA alone, AI-EA alone and cEA were significantly lower than the sham-EA group. Similar to hs-CRP results, in the rats treated with cEA, positive interaction effect was observed between SI-EA and AI-EA compared to either SI-EA or AI-EA alone in lowering IL-6 levels (*P* < 0.05 for interaction effects, Table 4).

**Table 4:** Concentrations of IL-6 after 5 weeks of repetitive SI-EA, AI-EA, cEA (i.e., SI-EA plus AI-EA), and sham-EA treatment in DSSH rats.

IL-6 (pg/ml)				
		SI-EA		Margin
		Yes	No	
AI-EA	Yes	16.6 ± 2.4*^,^**	26.9 ± 5.9*	21.8 ± 3.3
	No	32.5 ± 4.4*	70.0 ± 10.4	51.3 ± 7.3
Margin		24.5 ± 3.2	48.9 ± 8.0	

Values are means ± SE. IL-6, interleukin 6. Two-way ANOVA analysis indicates that SI-EA interacts positively with AI-EA in lowering IL-6 concentration.

*P < 0.05, compared to sham EA.

**P < 0.05, cEA compared to AI-EA or SI-EA.

## Discussion

The effectiveness of EA in treating hypertension has produced mixed outcomes in previous studies, possibly due to lack of understanding of the underlying mechanisms and the rationale for stimulating specific acupoints to achieve the intended effect. To address this issue, we conducted the present study that investigated the effects of EA stimulation at different acupoints that have demonstrated differential effects on sympathoexcitation and inflammation, both separately and in combination, on BP reduction as well as in modulating biomarkers that support the underlying biological pathways. In the present study, we found that anti-inflammatory EA treatment at SP6-7 acupoints mitigated progression of BP elevation over time compared to the sham-EA treated rats but had milder effect on absolute BP reduction. Sympathoinhibitory EA treatment at ST36-37 acupoints for five weeks significantly reduced BP and had a greater absolute BP reduction than AI-EA. Combining AI-EA and SI-EA during cEA treatment yielded the highest effect on absolute reduction in SBP and DBP, with reduction of BP toward normotensive levels compared to SI-EA or AI-EA alone. A positive interaction between AI-EA and SI-EA during cEA treatment was observed.

SI-EA and cEA, but not AI-EA, reduced sympathetic tone as indicated by a reduction of plasma NE levels relative to sham-EA treated rats. cEA reduced plasma concentrations of hs-CRP and IL-6 greater than SI-EA or AI-EA alone, again with a significant and positive interaction. Collectively, these findings from the present 2 × 2 factorial design study suggest that cEA therapy produces greater decreases in biomarkers of elevated SNA and chronic inflammation than either SI-EA or AI-EA alone, which potentially underlies the greater reduction in elevated SBP and DBP seen in hypertensive animals that received cEA treatment.

Although over 20 acupoints have been assessed in clinical studies, many have shown minimal impact on cardiovascular regulation centers in the brain in preclinical studies (11, 14, 17). Over the past two decades, we have conducted a series of pre-clinical mechanistic studies on the effects of acupuncture on reflex-induced increased BP and sustained hypertension (41, 42). Using a standardized approach, we have found that low-frequency (2 Hz) and low-intensity EA at acupoints ST36-37 and/or P5-6 significantly lowers hypertension or reflex-induced elevated BP by inhibiting sympathetic neuronal activity, which is similar to direct electrical stimulation of the median nerve and/or deep peroneal nerve (13, 23, 27, 43–45). Previous studies also have demonstrated that acupuncture stimulation of the acupoints ST36-37 provides substantial input to the rVLM, which essentially modulates the elevated sympathetic tone (22, 27, 46). Additionally, acupuncture stimulation at specific acupoints SP6-7, which overlie the tibial nerve, reduces inflammation in sepsis and arthritic conditions (39, 40). Based on these mechanistic results, EA at acupoints ST36-37 was chosen as SI-EA treatment, while EA at acupoints SP6-7 was selected as AI-EA treatment in the present study to test our hypotheses.

Elevated sympathetic activity concurrent with high levels of NE concentration are commonly observed in individuals with HTN (47–49). In our present study, we observed increased NE levels in DSSH rats treated with sham-EA, which is consistent with previous research. Moreover, we have shown that EA at acupoints ST36-37 reduces sympathetic outflow and blood pressure through rVLM opioids in cold-induced and reflex-induced hypertensive animals (22, 27, 46), while the effect of SI-EA on elevated SNA in DSSH rats remains unclear. In the present study, we investigated the effects of repetitive SI-EA treatment at acupoints ST36-37 on elevated sympathetic activity in DSSH rats. Our findings suggest that this treatment significantly reduces elevated SNA, which is evidenced by a reduction in plasma NE concentration in the rats. Rats that received AI-EA treatment did not experience changes in blood NE concentrations or demonstrate a significant interaction with SI-EA, indicating that AI-EA treatment did not contribute to reduction in sympathetic activity.

Chronic low-grade inflammation, characterized by increases in proinflammatory biomarkers such as IL-6 and hs-CRP, contributes to the development of essential hypertension (50–53). Increases in IL-6 and hs-CRP predict incidence of hypertension in older individuals without cardiovascular disease (7, 54). In our present study, we observed that inflammatory biomarkers such as hs-CRP and IL-6 were increased in the DSSH rats that received sham-EA treatment. Previous studies have demonstrated that targeting inflammation may be effective in improving BP in individuals with essential HTN (50–53). In our study, we found that repetitive AI-EA treatment significantly lowered blood hs-CRP and IL-6 levels in DSSH rats, which is well aligned with the anti-inflammatory effects of acupuncture at acupoints SP6-7 (9, 39, 40). We also observed that SI-EA treatment notably reduced blood hs-CRP and IL-6 levels in DSSH rats, which is supported by previous research in which acupuncture at ST36-37 decreases proinflammatory cytokines (55–57). Considering the effects of SI-EA and AI-EA on SNA together with anti-inflammation, we have noted that SI-EA treatment decreases both elevated sympathetic activity and inflammation, while AI-EA only reduces inflammation. This difference may explain the effect size of BP-lowering between SI-EA and AI-EA treatment. Our observation implies that repetitive SI-EA alone attenuates the progression of moderate HTN to severe HTN and further reduces it below the pre-EA treatment levels through reduction in both sympathetic activity and inflammation, whereas AI-EA treatment can prevent progression of moderate HTN to severe HTN via reduction in inflammation but fails to decrease BPs below the levels observed before EA treatment.

Chronic low-grade inflammation, characterized by increases in proinflammatory biomarkers such as IL-6 and hs-CRP, contributes to the development of essential hypertension (50–53). To address this issue, we hypothesized that targeting both the sympathetic and immune system in a multi-modal approach would result in greater BP reduction than targeting either system alone in an interactive fashion. We selected two sets of specific acupoints, ST36-37 and SP6-7, based on the mechanisms involving neuronal and inflammatory pathways. Our approach is in contrast to the stimulation of multiple acupoints without consideration of underlying mechanism, which could potentially result in negative or neutral effects when combined (11, 14, 16, 17). In our present study, we used a 2 × 2 factorial randomized design to allocate DSSH rats to receive combined SI-EA and AI-EA (i.e., cEA), SI-EA alone, AI-EA alone, or sham-EA treatments, allowing us to evaluate the individual and combined effects of the treatments on blood pressure and underlying mechanisms measured with sympathetic biomarkers NE, and inflammatory biomarkers hs-CRP and IL-6 in plasma (8, 13, 58, 59). Our findings indicate that repetitive cEA treatment using both sets of acupoints resulted in greater reductions of systolic and diastolic BP than either SI-EA alone or AI-EA alone. This observation is the result of SI-EA interacting positively with AI-EA affecting blood pressure, as well as on the modulation of sympathetic outflow and inflammatory responses. Previous studies support the use of factorial trials for evaluating two interventions (58, 60, 61). Altogether, our mechanistic approach suggests that cEA treatment may be a more effective method of reducing BPs in hypertensive patients than targeting either sympathetic or immune system alone.

**Figure 4:**
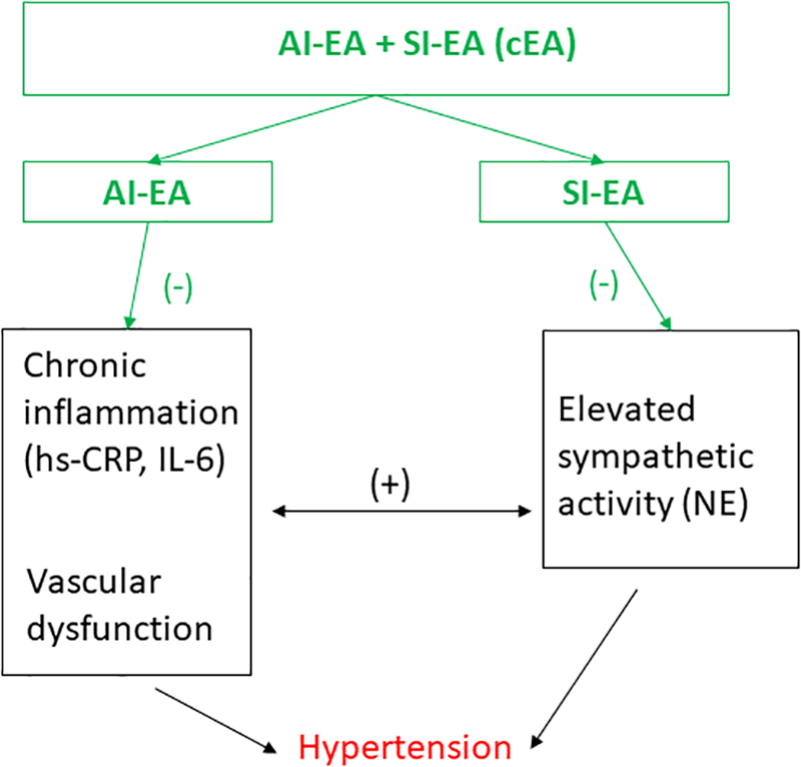
Schematic diagram summarizes the effects of electroacupuncture (EA) on hypertension and their potential underlying mechanisms. Anti-inflammatory EA (AI-EA) treatment slows down the advancement of high blood pressure primarily through the alleviation of chronic inflammation. On the other hand, Sympathoinhibitory EA (SI-EA) significantly lowers blood pressure by inhibiting sympathetic activity. Meanwhile, combining EA (cEA) treatment demonstrates a more substantial reduction in hypertension by modulating the intricate interplay between elevated sympathetic activity and inflammatory mediators. (−), decrease; (+), increase. NE, norepinephrine; hs-CRP, high sensitive C-reactive protein, and IL-6, interleukin 6.

To summarize, essential HTN involves multiple pathways including autonomic nervous and immune systems. Our findings illustrate that the development of HTN is associated with increases in both sympathetic tone and inflammatory mediators such as hs-CRP and IL-6 in the DSSH rats that received sham-EA treatment, suggesting that this rodent model of hypertension is similar to that observed in essential hypertensive patients. The interconnection of these two systems underscores the complex interaction between elevated sympathetic activity and inflammatory mediators in HTN. The present study is the first study to reveal that combined EA treatment (SI-EA and AI-EA) led to greater reduction of high SBP and DBP than individual SI-EA or AI-EA, through reductions in both sympathetic tone and inflammatory mediators as well as their positive interactions, as depicted in Figure 4. The effect size observed from cEA suggests that cEA can be a viable non-pharmacological option to treat clinical hypertension, as the pathological processes observed in the DSSH rat model similar to those occurring in essential hypertensive patients.

Future studies are warranted to explore the effects of cEA treatment on neuroinflammation specifically in the central autonomic nervous regions in hypertensive subjects, and to examine whether the interaction we observed between sympathetic tone and inflammation broadly can be traced to possible reduction in neuroinflammation and pre-sympathetic neuron activity in the central cardiovascular regions.

## Materials and methods

### Animals and hypertension development

**Figure 5:**
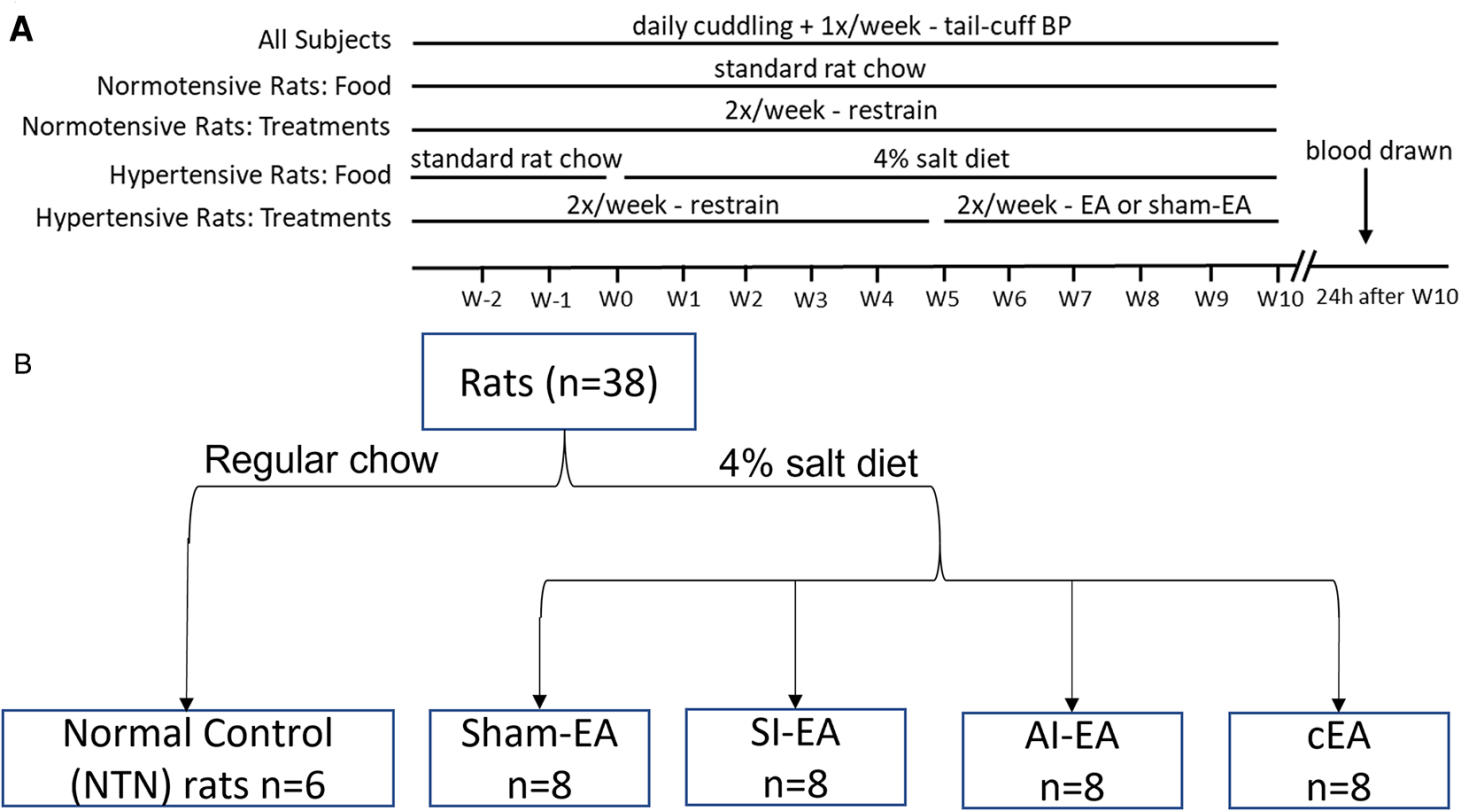
Timeline and group assignments of the current study. Timeline of experiments shown in (**A**). Dahl salt-sensitive (DSS) hypertensive rats were randomly allocated into SI-EA, AI-EA, cEA, or Sham-EA groups following a 2 × 2 factorial design. These rats were fed with 4% NaCl (high salt) diet for 10 weeks to develop hypertension. They were treated with repetitive EA or Sham-EA for 30 min twice weekly for five weeks once the blood pressures (BP) of these rats reached moderate hypertensive range. Blood pressures were evaluated by tail-cuff weekly in all 5 groups of rats. One of the 5 groups of DSS rats was fed with standard rat chow and their BP remained in normotensive range. At the end of the experiment, plasma samples were collected from the heart of each rat under anesthesia. (**B**) displaying flow diagram of rats assigned to different EA regimen groups.

A total of 38 young adult male Dahl salt-sensitive (DSS) rats (5–6 weeks old) were purchased from Charles River Laboratories, Inc. These rats were housed at room temperature (25^o^C) with tap water ad libitum and kept under a 12 h/12 h light/dark circle during the study (i.e., 13 weeks duration). Figure 5 displays the timeline of experiments. All experimental animal procedures were reviewed and approved by the Institutional Animal Care and Use Committee of the University of California Irvine. The study conformed to the American Physiological Society's “Guiding Principles in the Care and Use of Animals.” After five days of acclimatization at UCI, we provided intensive care (15 min/day) in handling and training the rats to ensure minimal stress and discomfort throughout the experiment. All DSS rats were fed with standard rat chow (0.4% NaCl diet) for the first two weeks, then 32 of the rats were switched to a 4% NaCl (high salt) diet for the next 10 weeks (Figure 5, experimental timeline) and 6 of the rats remained on standard chow serving as the normotensive controls.

### Blood pressure and heart rate measurements

Digital BP and HR data in conscious rats were recorded non-invasively with a volume pressure recording sensor and an occlusion tail-cuff (CODA System, Kent Scientific) weekly following the CODA system instruction and previous description (22, 62). Briefly, 10 days prior to recording BP and HR, conscious rats were familiarized with the procedures for tail-cuff BP monitoring, including warmth and restraint. On experimental day, each rat was cuddled for 15 min for stress reduction prior to being placed into a warmed restrainer for tail-cuff BP recording. Digital BP and HR values were recorded. BPs and HRs in all rats were evaluated weekly (Figure 5A and Methods in Supplement).

### EA treatment in conscious DSS hypertensive rats

The DSS rats fed with the high salt diet developed moderate hypertension (SBP within 160 ± 5 mmHg) at Week 5. The rats were then allocated randomly into groups of SI-EA (ST36-37), AI-EA (SP6-7), combined SI-EA and AI-EA (cEA), or sham-EA according to a 2 × 2 factorial design (Figure 5B). After rats were trained to become accustomed to the EA treatment procedure (Supplement Materials), starting from Week 5, acupuncture needles were inserted into acupoints ST36-37 (SI-EA group), SP6-7 (AI-EA group), or both sets of acupoints (cEA) for 30-min EA treatment twice weekly. The ST36-37 acupoints are located 5 mm below the knee joint of the hind limb and 2 mm lateral to the anterior tubercle of the tibia (overlying deep peroneal nerve) (27, 63). The SP6-7 acupoints are located 3 mm proximal to the upper border of the medial malleolus, between the posterior border of the tibia and the anterior border of the Achilles tendon (overlying the tibial nerve) (64). Locations of the ST36-37 and SP6-7 acupoints anatomically are analogous to those in humans (65). The EA application (2 Hz, 0.1–0.4 mA, 0.5 ms duration) was generated by a current stimulator that was connected to an isolation stimulus unit (Grass, Model S88) to provide consistent electrical stimulation (2 Hz, 0.1–0.4 mA, 0.5 ms duration) for 30 min EA treatment twice weekly for five weeks (Figure 5A). Previously, we observed that 5 weeks of EA application at ST36-37 acupoints for 30 min twice weekly alleviates cold-stress induced hypertension (22). In rats allocated to sham-EA, needles were inserted at acupoints ST36-37 and acupoints SP6-7, but without the delivery of electric current. Correct insertion of needles at acupoints ST36-37 or SP6-7 was confirmed by observation of repetitive flexor twitches of the paws (ST36-37) or sideway twitches of the halluces (SP6-7). These slight movements indicate that EA application at ST36-37 also activates the motor fibers in the mixed nerve bundle comprised of the deep peroneal nerve, while EA at SP6-7 stimulates the motor fibers in the tibial nerve (66, 67). We have shown that motor nerve stimulation does not participate in the EA-modulation of cardiovascular responses (24, 68). The 6 normotensive (NTN) rats received 30-min restraining sessions twice weekly throughout the experiments.

We have conducted preliminary studies determining the effect of cEA on BP in DSS normotensive rats. In 4 NTN rats fed with standard chow, cEA treatment for five weeks similar to the timeline shown in Figure 5A did not change their BPs (SBP, 130.8 ± 0.8 vs. 130.4 ± 1.5 mmHg, DBP, 89.2 ± 1.4 vs. 88.9 ± 1.7 mmHg, pre-EA vs. post-EA). These data were supported by our previous studies in which we have documented that EA treatment has little or no influence on BP in normotensive humans or animals (25, 27, 69). Thus, the present study focused on the experimental protocols that examine the effects of the 4 different EA regimens on the DSS hypertensive rats.

### Plasma biomarkers measurement

*Plasma Sample Preparation*. Each rat was anesthetized with ketamine (60 mg/kg, i.p.) 24 hours after the end of experiment (Figure 5A). Blood sample (4 ml) was collected from the heart and placed into a tube that contains 3.8% ethylenediaminetetraacetic acid (EDTA). Next, the anti-coagulated blood was centrifuged (Beckman, Model Allegra 6R) at 1,500 g for 15 min and then the plasma was removed and stored in aliquot in several small tubes at −80^o^C for later use.

*Analysis of plasma norepinephrine (NE).* Concentration of plasma NE was evaluated in duplicate using commercially available comparative ELISA kits (My BioSource, San Diego, USA). The NE was detected at 450 nm with a microplate reader (Molecular Devices, USA) according to the manufacturer's instructions. The sensitivity of this assay was 9.4 pg/ml with a <8% intra-assay and <10% inter-assay coefficient of variability.

*Analysis of plasma inflammatory markers.* Levels of plasma hs-CRP and IL-6 were determined quantitatively in duplicate using commercially available sandwich ELISA kits (Invitrogen, USA). The hs-CRP and IL-6 were measured at 450 nm with a microplate reader (Molecular Devices, USA) according to the manufacturer's instructions. The hs-CRP assay displayed a sensitivity of 0.2 ng/ml with a <10% intra-assay and <12% inter-assay coefficient of variability. The IL-6 assay exhibited a sensitivity of 0.3 pg/ml with a <7% intra-assay and <8% inter-assay coefficient of variability.

### Statistical analyses

BP and HR as well as concentrations of biomarkers are presented as means ± SE. The present study used a 2 × 2 factorial design (Figure 5B). All data were normally distributed as determined by the Shapiro-Wilk test. The primary hypothesis of the present study was that the combination of SI-EA and AI-EA would lead to a greater reduction of BP than SI-EA or AI-EA alone.

Analysis of this hypothesis was conducted using two-way ANOVA followed by the Holm-Sidak test with four EA regimens and the interactions in their effects. This analysis also was performed for evaluating the effects of four EA regimens on the concentrations of NE, hs-CRP, and IL-6. Next, when the two-way ANOVA analysis indicates the presence of interactions, further analyses were carried out “inside the table”; while in the absence of interactions, further analyses were carried out “at the margins” (58, 59).

Additionally, one-way repeated ANOVA followed by the Tukey test was used to compare the weekly-dynamic changes in BPs and HRs during the 5-week treatment in each group. A Students' t-test was used to compare plasma NE, hs-CRP, and IL-6 between any two groups. A *P*-value < 0.05 was considered statistically significant. All statistical analyses were performed with SigmaPlot software (Systat Software Inc., USA).

## Data Availability

The original contributions presented in the study are included in the article/**Supplementary material**, further inquiries can be directed to the corresponding author/s.
