# Supplementary material for: Sympathoinhibitory electroacupuncture (EA) interacts positively with anti-inflammatory EA alleviating blood pressure in hypertensive rats
Source: Front Cardiovasc Med. 2023 May 30;10:1140255. doi: 10.3389/fcvm.2023.1140255 (PMC10262041; doi:10.3389/fcvm.2023.1140255)
Supplement: Supplementary file 1 [file Datasheet1.docx]

Supplementary Material

Sympathoinhibitory Electroacupuncture (EA) Interacts Positively with Anti-Inflammatory EA Alleviating Blood Pressure in Hypertensive Rats

Liang-Wu Fu*, Yiwei D. Gong*, Anh T. Nguyen, Zhi-Ling Guo, Stephanie C. Tjen-A-Looi, Shaista Malik

*** Correspondence:** Liang-Wu Fu: [lwfu@hs.uci.edu](mailto:lwfu@hs.uci.edu)

# Supplementary Methods

Animal Handling and Acclimatization

To acclimatize each rat to the laboratory environment, 2 rats per cage were placed in the laboratory with IACUC approval for 5 days (8 hours daily). Within 8 days of arrival, each rat was cuddled gently for 15 min daily to familiarize them with handling.

Training of Rats for Tail-Cuff BP Measurement

For 10 days prior to recording BP and HR, each conscious rat was familiarized with the procedures for tail-cuff BP monitoring, including warmth and restraint. According to our observations in preliminary studies, the stress in rats was minimized by familiarizing rats with regular handling and all steps of the tail-cuff procedure including placing rat into a restrainer and placement of the O and V cuffs on the tail, thus permitting the technical goal of quality BP measurements. 10 days were chosen as the optimal rat training period according to our observation that by Day 9-10, rats were accustomed to the procedure. On the experimental days per week, each rat was cuddled for 15 min to sustain calmness and minimize stress prior to being placed into a restrainer and heated in the normal manner for tail-cuff BP recording (CODA System, Kent Scientific). The first five inflations and deflations were used to condition and acclimatize the rats to the measurement procedures. After stabilization, digital BP and HR values were recorded, and at least 8 BP and HR data accepted by the CODA 3.0 program were taken to acquire an average BP and HR. For each set of tail-cuff BPs and HRs measurements/rat, data that fell out of the range of Mean ± 2SD (i.e., standard deviation) were excluded from data analyses. Figure 5 outlines the timeline schedule for measuring BPs and HRs in all rats twice a week. The BP data were then averaged weekly and presented in Figure 1. To provide a timely representation of the rats’ BP status from -2 week to 0 week prior to the commencement of the high salt diet, the BP data were presented twice per week.

Training of Rats to become accustomed to acupuncture procedures.

Prior to the application of EA, conscious rats were trained for 2 weeks (i.e., from Week 3 to 4) to become accustomed to handling and EA procedures. Rats were gently restrained for 30-min twice weekly in a sling that wrapped the body of a rat excluding the four limbs. This procedure safely and effectively immobilized the rats. During the restraining session, acupuncture needles (0.16 mm) were inserted into and left in the muscle located at the back of their hind legs for 30 min to train rats to become complacent to EA needling. Two weeks were chosen as the optional rat training period based on the observation that by Day 13 to 14, rats were sufficiently compliant with the procedure.

# Supplementary Figures

**Supplementary Figure 1.** **Dynamic or weekly changes of heart rate (HR) in the Dahl salt-sensitive hypertensive (DSSH) rats treated with repetitive electroacupuncture (EA) and in DSS normotensive (NTN) rats.** The 4 EA regimens include sympathoinhibitory EA (SI-EA), anti-inflammatory EA (AI-EA), combined SI-EA and AI-EA (cEA), and sham-EA. Data are expressed as mean ± SE.

AAA


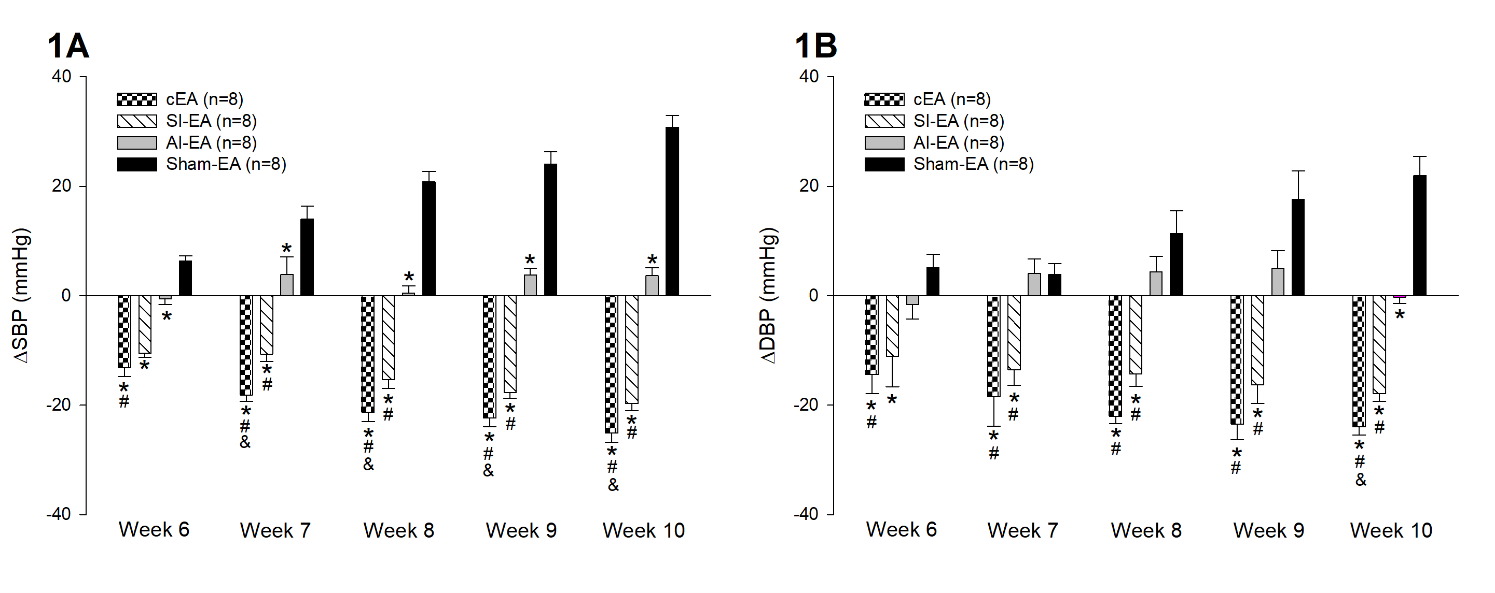


B

**Supplementary Figure 2.** **Bar histograms showing weekly changes of systolic blood pressure (SBP, Panel A) and diastolic blood pressure (DBP, Panel B) at week 6 to week 10 in rats treated with cEA, SI-EA, AI-EA, and sham-EA.** ΔSBP is equal to the difference in SBP between weeks 6 and 5, 7 and 5, 8 and 5, 9 and 5, and 10 and 5. ΔDBP is equal to the change in DBP from weeks 5 to 6, 7, 8, 9, or 10. *, P<0.05, active EA vs. sham-EA; #, P<0.05, cEA or SI-EA vs. AI-EA; and &, P<0.05, cEA vs. SI-EA.
